# Paper Versus Digital Data Collection for Road Safety Risk Factors: Reliability Comparative Analysis From Three Cities in Low- and Middle-Income Countries

**DOI:** 10.2196/13222

**Published:** 2019-05-28

**Authors:** Amber Mehmood, Niloufer Taber, Abdulgafoor M Bachani, Shivam Gupta, Nino Paichadze, Adnan A Hyder

**Affiliations:** 1 International Injury Research Unit Department of International Health Johns Hopkins Bloomberg School of Public Health Baltimore, MD United States; 2 Milken Institute School of Public Health The George Washington University Washington, DC United States

**Keywords:** information technology, public health informatics, mHealth, risk factors, population surveillance

## Abstract

**Background:**

Rapid advances in mobile technologies and applications and the continued growth in digital network coverage have the potential to transform data collection in low- and middle-income countries. A common perception is that digital data collection (DDC) is faster and quickly adaptable.

**Objective:**

The objective of this study was to test whether DDC is faster and more adaptable in a roadside environment. We conducted a reliability study comparing digital versus paper data collection in 3 cities in Ghana, Vietnam, and Indonesia observing road safety risk factors in real time.

**Methods:**

Roadside observation of helmet use among motorcycle passengers, seat belt use among 4-wheeler passengers, and speeding was conducted in Accra, Ghana; Ho Chi Minh City (HCMC), Vietnam; and Bandung, Indonesia. Two independent data collection teams were deployed to the same sites on the same dates and times, one using a paper-based data collection tool and the other using a digital tool. All research assistants were trained on paper-based data collection and DDC. A head-to-head analysis was conducted to compare the volume of observations, as well as the prevalence of each risk factor. Correlations (*r*) for continuous variables and kappa for categorical variables are reported with their level of statistical significance.

**Results:**

In Accra, there were 119 observation periods (90-min each) identical by date, time, and location during the helmet and seat belt use risk factor data collection and 118 identical periods observing speeding prevalence. In Bandung, there were 150 observation periods common to digital and paper data collection methods, whereas in HCMC, there were 77 matching observation periods for helmet use, 82 for seat belt use, and 84 for speeding. Data collectors using paper tools were more productive than their DDC counterparts during the study. The highest mean volume per session was recorded for speeding, with Bandung recording over 1000 vehicles on paper (paper: mean 1092 [SD 435]; digital: mean 807 [SD 261]); whereas the lowest volume per session was from HCMC for seat belts (paper: mean 52 [SD 28]; digital: mean 62 [SD 30]). Accra and Bandung showed good-to-high correlation for all 3 risk factors (*r*=0.52 to 0.96), with higher reliability in speeding and helmet use over seat belt use; HCMC showed high reliability for speeding (*r*=0.99) but lower reliability for helmet and seat belt use (*r*=0.08 to 0.32). The reported prevalence of risk factors was comparable in all cities regardless of the data collection method.

**Conclusions:**

DDC was convenient and reliable during roadside observational data collection. There was some site-related variability in implementing DDC methods, and generally the productivity was higher using the more familiar paper-based method. Even with low correlations between digital and paper data collection methods, the overall reported population prevalence was similar for all risk factors.

## Introduction

### Background

Road traffic injuries (RTIs) are the ninth leading cause of death worldwide, primarily affecting the young and productive age groups. In addition to causing over 1.35 million deaths each year, road traffic crashes are responsible for 50 million nonfatal injuries [1]. Although the rapid economic growth seen in many low- and middle-income countries (LMICs) has raised living standards and led to a reduction in many diseases of poverty, increased motorization without an increase in traffic enforcement or improvements in road environments has led to a rise in motor vehicle crashes [2-5]. Consequently, LMICs share a larger proportion of deaths and disability, and it is estimated that 966 to 1160 per 100,000 population disability-adjusted life years are lost because of RTIs [6].

To tackle this international problem, the Global Plan for the Decade of Action for Road Safety 2011-2020 recommended a range of road safety measures, including improvement in road user behaviors as an important pillar [7]. These recommendations focus on the development and implementation of comprehensive programs and strategies to positively affect seat belt and child restraint use, correct helmet wearing, speeding, and drunk driving. The Bloomberg Initiative for Global Road Safety (BIGRS), a consortium of international partners funded by Bloomberg Philanthropies, promotes the adoption of internationally recognized best practices to improve these 4 risk factors in 10 selected cities from across 9 LMICs [8-10]. As part of the project, the Johns Hopkins International Injury Research Unit conducts semiannual observational studies with local partners to measure the prevalence of these 4 RTI risk factors over time: helmet use, seat belt use, speeding, and drunk driving. These data provide evidence to inform program development, as well as monitoring and evaluation of interventions in the project.

### Roadside Observational Studies for Risk Factor Monitoring

Paper-based data collection has been the standard method for primary roadside observational studies, and until recent years, the only method [11,12]. Paper-based data collection methods have certain advantages that make them easily adaptable. Paper-based tools are more flexible, immediately deployable, and do not require any specialized training other than the content of questionnaire. Writing on paper forms is easier especially for short and concise questionnaires. However, as other researchers have observed, legibility related errors are frequent, storage costs can be prohibitive, deployment and tracking of surveys are challenging, and double data entry is expensive and time-consuming [13,14]. Sometimes, when using paper data collection methods, other forms of data, including photographs or Global Positioning System (GPS) coordinates, require separate equipment and careful tracking to link external data to the correct observation. Moreover, in administered questionnaires, there may be variability among surveyors in adhering to complex eligibility requirements or logical dependencies (skip patterns) across questions [15].

Recently, to facilitate real-time data collection in a roadside environment, mobile health (mHealth) tools were developed for population-level observational studies on 3 road safety risk factors, speeding, helmet use, and seat belt use, as a part of the BIGRS project. Electronic (digital) methods of data collection have merged the process of data collection and data entry, potentially saving costs and time [16]. The diversity of mHealth apps has generated immense interest among researchers to test innovative ideas, especially where accuracy and standardized data collection is required. Transitioning from traditional paper-based methodology into digital data collection (DDC) supports rapid aggregation and analysis of a large amount of data by avoiding the costs and time lag of data entry [15]. In addition, DDC also facilitates remote monitoring of the data collection process and can improve data quality by introducing standardized responses, skip patterns, logic checks, and automatic calculations [17-19]. However, these advantages of DDC must be compared with the productivity and reliability of widely accepted and established paper-based data collection method.

### Rationale and Study Objectives

Although DDC is rapidly replacing traditional paper-based methods, the majority of studies or commentary about DDC method do not use multiple methods to establish its reliability by independent data collectors on the same targets and are usually done in the context of household surveys rather than observational studies [14,20,21]. Owing to lack of comparative studies, there is little evidence about the productivity, reliability, and efficiency of large volume data collection using mobile devices in a highly dynamic roadside environment [20]. The term *productivity* refers to sheer quantity, and in the context of observational studies, the amount of data collected per session, real time in the field, distinguishing it from *efficiency*, which is used in the context of quality of data that might include creating output in less time, using fewer resources, or spending less money. In the context of project management, efficient output may have different dimensions, including human and material cost, effort, turnaround time, etc. Reliability of a new tool or method indicates its ability to produce same or consistent results when compared with a reference or standard method. This study aimed to assess productivity and reliability of DDC by comparing simultaneously collected paper and digital data in terms of volume of observations, overall measurement of prevalence of road safety risk factors, and interobserver agreement about a busy roadside environment. The paper then discusses the impact of study findings in guiding the choice between paper and digital methods of data collection in different contexts.

## Methods

### Setting

The study was conducted in 3 different cities where roadside observational studies were taking place: Accra, Ghana; Bandung, Indonesia; and Ho Chi Minh City (HCMC), Vietnam. These cities were selected out of the 10 participating in the BIGRS project, based on the willingness of the local partners to switch from paper to digital format. Simultaneous paper-based data collection and DDC were conducted in Accra during March 2017, in HCMC during April 2017, and in Bandung during August 2017, as part of a routine semiannual data collection schedule.

The BIGRS Project team used KoBoToolbox data collection software and its KoBoCollect Android smartphone application, which was developed by the Harvard Humanitarian Initiative as an open source suite of tools for data collection and analysis [22]. The digitization process included programming digital forms to be downloaded on mobile app, using Android tablets for data collection and uploading information to the secure cloud server [23]. DDC forms for each risk factor were based on the same tools used in paper-based data collection method. To maintain standardization and quality, all the digital forms were the same, though the forms were available in English for Accra and were bilingual for HCMC (Vietnamese and English) and Bandung (Indonesian and English).

In all 3 cities, local data collectors were hired and trained over 2 days to familiarize them with the study protocols, as well as the Android environment and the KoBoCollect app, including mobile data entry process, saving digital forms, and uploading data to the server. All data collectors were trained on both digital and paper data collection methods. This training session was followed by a mandatory hands-on practice for both digital and paper data collectors. Similarly, supervisors and data managers were trained to manage field site data collection, monitor data upload and server activity, and download data from the server. Any issues with data entry, saving, and uploading were identified and resolved during training and practice, before the roadside observational studies.

### Data Collection Protocol

Within each city, locations for observation were selected using stratified randomization to ensure that all major road types and city administrative divisions were represented. Standardized observation methods were employed across all observation sites. At each location, observations were done by 2 independently working teams on the same date and times with one team using paper-based forms and the other used digital forms. Each team consisted of 1 observer who viewed vehicles and conveyed the information to the data recorder who marked the presence of risk factors and demographic data for each vehicle as applicable. Data collectors were randomly rotated among teams and between digital and paper data collection methods throughout the study to avoid individual data collector competence influencing the productivity or reliability of data collection method. Each data collector’s schedule was randomly varied by date, time, location, data collection partner, and data collection method (paper or digital). Observations at each location were done during both weekdays and weekends and both rush hours and off-peak hours.

For speeding assessments, the sites were carefully chosen to avoid junctions or intersections, or areas where vehicles were slowing down because of construction or road blocks, as well as entrances to parking lots, gas stations, malls, or shopping centers. For observations on seat belts and child restraint use, junctions, intersections, or entrances of gas stations and rest areas where vehicles travel at reduced speeds were selected to facilitate close observations and ensure accuracy. The protocol required that only vehicles traveling in 1 direction were observed. Starting the observation with the vehicle closest to the curb or roadside also allowed data collectors to observe as many vehicles as possible with accuracy in a high-volume traffic flow.

At the beginning of the session at each site, the team filled a site description form using their respective method (digital or paper). These forms captured data about the road and traffic environment at each time, date, and location, including the traffic volume during a 15-min period, the weather, and the presence and nature of law enforcement, including the presence and placement of police and/or cameras for enforcement. Speeding observations also captured the posted speed limit and the existence of various environmental traffic calming measures, such as speed bumps. Data captured about the vehicle included the vehicle type (sedan, sport utility vehicle, truck, etc), vehicle ownership (private, commercial, government, etc), and for speeding observations, the actual speed of the vehicle in kilometer per hour (km/h). Finally, information on the vehicle’s occupants was captured during assessments of seat belt and helmet use, including each occupant’s gender, estimated age group, position within the vehicle, and use of safety equipment.

Consistent procedures and definitions were maintained between methods and across observation sites to ensure comparability of results. The metric for comparisons was volume of observations and prevalence of risk factor per 90-min session. We did not use an a priori number of observations and track the time to accomplish them. The helmet use risk factor was defined as wearing a strapped, standard helmet (not a cap helmet). Seat belt use was defined as wearing a buckled seat belt, or using a proper child restraint, on a single vehicle occupant. Speeding was defined as any speed in excess of the posted speed limit in kilometer per hour and levels of overspeed categorized in 5 or 10 km/h intervals.

### Statistical Analysis

Observation periods in digital and paper formats were matched to each other by date, time, and location; digital observation periods without corresponding paper observation periods were not included in the analysis, and vice versa. The few sessions that did not match were because of issues with logistics, staffing, or equipment.

A head-to-head comparison of digital and paper data collection was conducted to assess the productivity during each session matched by date, time, and location. After pooling all sessions, the mean number of observations were compared between paper and digital methods. In addition, 2 sample tests of proportions were used to compare overall helmet use and seat belt use prevalence, pooling across all observation sessions. Furthermore, chi-square tests of independence were used to evaluate whether the numbers of vehicles in different categories of overspeeding varied between digital and paper data collection methods, again pooling across all observation sessions [24]. This was done to assess whether the larger picture of traffic safety was the same between digital and paper data collection methods, despite any differences between individual digital and paper observation sessions.

The reliability of a method could be measured in terms of inter- or intraobserver variations for which *r*, interclass correlations, and kappa are appropriate statistics. In this study interrater reliability between digital and paper data collection methods were assessed using Pearson correlations for continuous measures and kappa for roadside environment data recorded as categorical variables, with the observation session used as the rating object [25,26]. Pearson correlation is appropriate for proportions when the majority of proportions are not close to 0 or 1. Although Spearman rank correlation is often used for proportions, we were interested in exact values rather than rank ordering and therefore Pearson correlation was more appropriate.

The risk factors were not pooled across the cities to better appreciate the contextual differences in the productivity and reliability of city teams.

Templates in Microsoft Excel were used for data entry from paper formats and all statistical analyses were conducted in STATA SE version 15.1 software package [27]. Ethical approval was obtained from the Institutional Review Board of Johns Hopkins Bloomberg School of Public Health, United States.

## Results

### Productivity in Digital and Paper Data Collection Methods

In Accra, there were 119 helmet use observation sessions matched exactly by date, time, and location between digital and paper data collection methods; 119 matched seat belt use observation sessions and 118 matched sessions observing speeding. In Bandung, each risk factor had 150 matched observation sessions. In HCMC, the numbers of matched sessions were somewhat lower, with 77 matched helmet use observation sessions, 82 matched seat belt use sessions, and 84 sessions matched for speeding observations. This lower number was because of both the fewer number of sessions conducted in digital and paper data collection methods and mismatches by date, time, or location between digital and paper observation sessions. As the number of sessions, and hence the total sample size for each risk factor varied by city, the mean volume of observations per session was used for comparison.

In addition to the number of sessions, the number of observations made per session varied among the 3 cities and across risk factors, with Bandung generally having higher productivity per session, followed by Accra, and last by HCMC (Table 1). The number of observations made per session was lower among research assistants conducting DDC as compared with paper data collection, with correlations between digital and paper for the same date, time, and location ranging from 0.23 to 0.95 across cities and risk factors (Table 1).

To assess the impact these differences between digital and paper data collections in productivity and overall sample size may have had on the precision of our estimates, we calculated the level of precision for the current digital data sample size (Table 2). We also calculated the sample sizes needed to estimate proportions to achieve a CI half-width of 0.01 and 0.005, based on the DDC proportion. With 1 exception, all digital and paper sample sizes were able to provide estimates within 1 percentage point, and in 6 out of 9 cases, the sample size was large enough to estimate proportions to within a half percentage point, thus eliminating any risk of sample size affecting the overall prevalence of risk factors. We did not find any risk factor in the cities where the paper sample size was able to estimate precision to one or one-half percentage point, but not the DDC.

**Table 1:** Volume of observations: reliability between digital and paper observations.

Risk factor and city		Digital observation, mean (SD)	Paper observation, mean (SD)	Correlation value (*r*)	*P* value
**Helmet**					
	Accra	181.81 (84.87)	196.86 (91.53)	0.95	<.001
	Bandung	353.97 (104.03)	509.81 (151.97)	0.56	<.001
	Ho Chi Minh City	210.45 (86.29)	249.17 (107.32)	0.23	.04
**Seat belt**					
	Accra	200.47 (71.20)	258.31 (96.73)	0.73	<.001
	Bandung	199.78 (71.35)	245.23 (88.06)	0.52	<.001
	Ho Chi Minh City	62.40 (30.09)	51.56 (28.45)	0.32	.003
**Speeding**					
	Accra	305.32 (90.42)	331.65 (94.83)	0.84	<.001
	Bandung	807.49 (261.23)	1092.08 (435.45)	0.78	<.001
	Ho Chi Minh City	228.27 (109.80)	225.24 (131.98)	0.77	<.001

**Table 2:** Level of precision and sample size requirements.

Risk factor and city		Digital observation: existing sample size	Paper observation: existing sample size	Digital observation: current level of precision	Sample size required for estimation within 1 percentage point (0.01)	Sample size required for estimation within 0.5 percentage point (0.005)
**Helmet**						
	Accra	28,719	30,983	0.005	8655	34,618
	Bandung	71,846	101,197	0.003	8378	33,512
	Ho Chi Minh City	20,842	26,419	0.006	7524	30,094
**Seat belt**						
	Accra	55,983	58,024	0.004	9553	38,211
	Bandung	50,391	57,758	0.004	8573	34,292
	Ho Chi Minh City	8205	5981	0.011	9567	38,268
**Speeding**						
	Accra	36,028	39,135	0.004	7132	28,526
	Bandung	121,123	163,812	0.002	4572	18,285
	Ho Chi Minh City	19,175	18,920	0.002	613	2452

### Interobserver Agreement between Digital and Paper Data Collection Methods

There were some discrepancies between how digital and paper research assistants recorded the presence of police and camera enforcement at each site. The values of calculated kappa statistics ranged from just under 0.51 up to 1.00, indicating moderate to perfect agreement. In Accra, all speeding observation sessions recorded that there was no police presence and no camera enforcement; although there was 100% agreement between digital and paper data collection methods, without any variation, kappa is undefined (Table 3).

**Table 3:** Interrater agreement between digital and paper data collection methods on law enforcement and environmental deterrents.

Risk factor and city		Kappa value	*P* value
**Helmet^a^**			
	Accra	0.51	<.001
	Bandung	0.72	<.001
	Ho Chi Minh City	0.50	<.001
**Seat belt^a^**			
	Accra	0.93	<.001
	Bandung	0.92	<.001
	Ho Chi Minh City	0.59	<.001
**Speeding^a^**			
	Accra	N/A^b^	N/A^b^
	Bandung	0.68	<.001
	Ho Chi Minh City	1.00	<.001
**Speeding^c^**			
	Accra	0.94	<.001
	Bandung	0.78	<.001
	Ho Chi Minh City	0.95	<.001

^a^Comparing police presence, camera enforcement, or both.

^b^N/A: not applicable; no sites were observed to have police or camera enforcement through digital or paper data collection; although there was 100% agreement between digital and paper data collection, without any variation, kappa is undefined.

^c^Comparing environmental speed deterrents, including speed bumps, cross walks, and stop signs.

### Prevalence of Risk Factors and Reliability between Digital and Paper Data Collection Methods

The prevalence of each of the 3 risk factors were assessed with moderate to high levels of reliability between digital and paper data collection methods. Accra showed the highest levels of reliability overall. For example, on average 63% to 66% of motorcycle occupants per session were observed to wear helmets in both digital and paper data collection methods, for a correlation of *r*=0.94 (*P*<.001; Table 4). Reliability between digital and paper data collection methods was similar for seat belt use and speeding; the correlation for seat belt use was 0.76 (*P*<.001) and 0.97 (*P*<.001) in speeding. Bandung also had similar levels of correlation between digital and paper data collection per observation session, with excellent reliability in helmet use and speeding observations (*r*=0.89, *P*<.001 and *r*=0.95, *P*<.001, respectively), and very good correlation between data collection methods in seat belt use observations (*r*=0.70, *P*<.001). HCMC had lower and nonsignificant correlations between digital and paper data collection methods in helmet and seat belt use observations (*r*=0.11, *P*=.36 and *r*=0.08*,* *P*=.46, respectively). Interestingly, speeding correlation in HCMC was almost perfect (*r*=0.999, *P*<.001).

**Figure 1:**
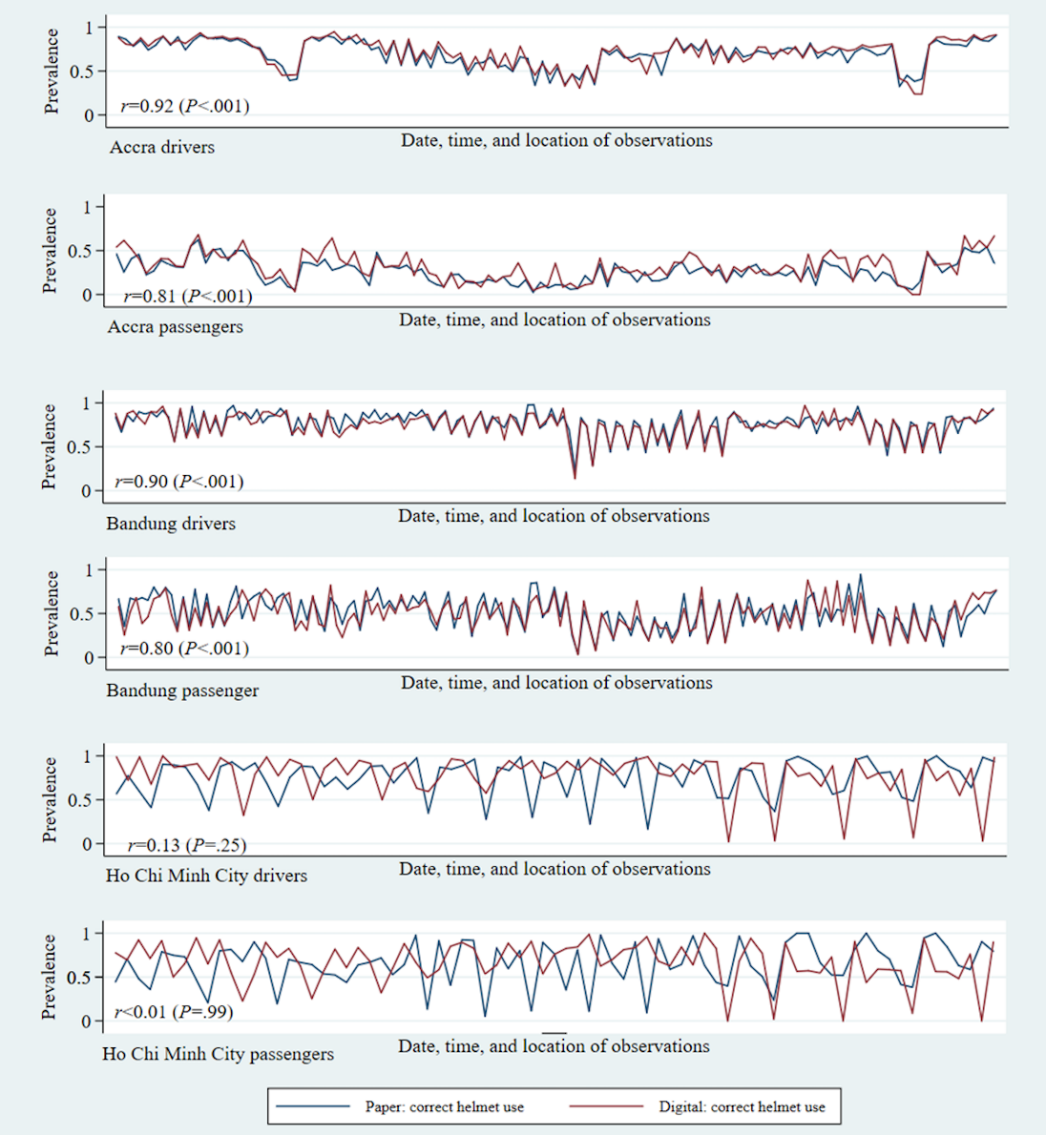
Prevalence of correct helmet use by occupant role: reliability between digital and paper observations.

**Figure 2:**
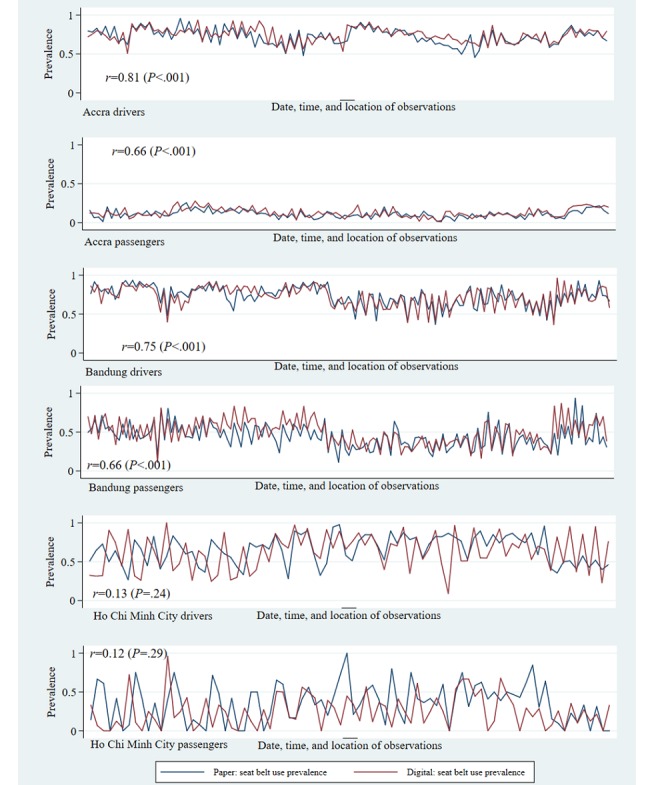
Prevalence of seat belt use by occupant role: reliability between digital and paper observations.

For helmet use and seat belt use risk factor assessments, reliability was also assessed within subgroups of road users, by gender, estimated age group, and occupant’s position within the vehicle. When further breaking down helmet and seat belt use by occupant’s role, driver or passenger, the correlations followed similar patterns as the overall figures, with Accra and Bandung showing very high levels of correlation, followed by HCMC (Figures 1 and 2). Across all 3 cities, observations made on drivers showed higher levels of reliability than observations made on passengers.

**Figure 3:**
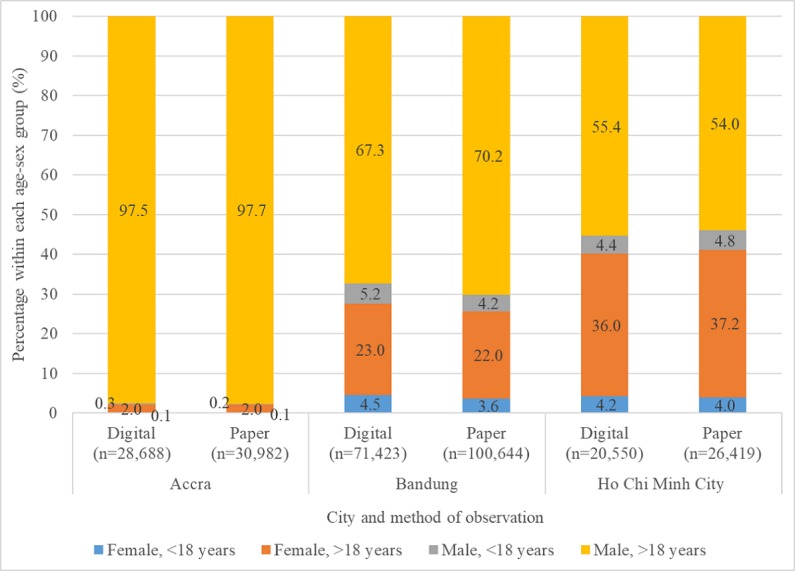
Motorcycle occupants observed by age-sex group.

In each city, the largest proportion of observed motorcycle occupants were males over the age of 18 years, with motorcycle occupants being almost exclusively adult males in Accra (98% of all occupants in both digital and paper data collection methods; Figure 3). Assessments of the seat belt use risk factor considered finer divisions of estimated age, as younger and older children should use different child restraints, rather than seat belts alone (Tables 5 and 6). Furthermore, the age groups and genders were estimated on best guess by data collectors, rather than exact ages and genders. However, as the gender of children is often difficult to assess, we have pooled the genders in the under 5 years and 6 to 11 years age categories.

**Table 4:** Prevalence of risk factors: overall proportions and 2 sample tests of proportions.

Risk factor and city			Digital proportion (SD)		Paper proportion (SD)		*P* value
**Helmet**							
	Accra	0.66 (0.47)		0.63 (0.48)		<.001	
	Bandung	0.68 (0.47)		0.70 (0.46)		<.001	
	Ho Chi Minh City	0.73 (0.44)		0.73 (0.45)		.18	
**Seat belt**							
	Accra	0.46 (0.50)		0.44 (0.50)		<.001	
	Bandung	0.63 (0.48)		0.66 (0.47)		<.001	
	Ho Chi Minh City	0.53 (0.50)		0.59 (0.49)		<.001	
**Speeding**							
	Accra	0.75 (0.43)		0.77 (0.42)		<.001	
	Bandung	0.14 (0.34)		0.12 (0.33)		<.001	
	Ho Chi Minh City	0.02 (0.12)		0.02 (0.12)		.65	

**Table 5:** Prevalence of helmet use by age-sex groups: reliability between digital and paper observations.

Risk factor and city		Digital observation proportion	Paper observation proportion	Correlation value (*r*)	*P* value
**Female, <18 years**					
	Accra	0.00	0.13	N/A^a^	N/A
	Bandung	0.21	0.20	0.35	<.001
	Ho Chi Minh City	0.44	0.50	−0.14	.23
**Female, >18 years**					
	Accra	0.26	0.25	0.33	<.001
	Bandung	0.61	0.63	0.79	<.001
	Ho Chi Minh City	0.70	0.72	0.17	.13
**Male, <18 years**					
	Accra	0.22	0.10	0.27	.14
	Bandung	0.35	0.32	0.37	<.001
	Ho Chi Minh City	0.51	0.48	0.15	.20
**Male, >18 years**					
	Accra	0.67	0.64	0.94	<.001
	Bandung	0.76	0.78	0.87	<.001
	Ho Chi Minh City	0.80	0.77	0.09	.42

^a^N/A: not applicable.

**Table 6:** Prevalence of seat belt use by age-sex groups: reliability between digital and paper observations.

Risk factor and city			Digital observation proportion		Paper observation proportion		Correlation value (*r*)		*P* value
**Both genders, <5 years**									
	Accra	0.13		0.14		0.63		<.001	
	Bandung	0.07		0.07		−0.004		.97	
	Ho Chi Minh City	0.00		0.00		N/A^a^		N/A	
**Both genders, 5-11 years**									
	Accra	0.11		0.05		0.23		.23	
	Bandung	0.17		0.10		0.22		.02	
	Ho Chi Minh City	0.10		0.00		N/A		N/A	
**Female, 12-17 years**									
	Accra	0.10		0.17		−0.19		.33	
	Bandung	0.33		0.30		0.16		.23	
	Ho Chi Minh City	0.27		0.05		−0.04		.84	
**Female, 18-24 years**									
	Accra	0.13		0.11		0.13		.40	
	Bandung	0.64		0.51		0.06		.47	
	Ho Chi Minh City	0.16		0.10		0.29		.01	
**Female, 25-59 years**									
	Accra	0.30		0.26		0.70		<.001	
	Bandung	0.68		0.61		0.58		<.001	
	Ho Chi Minh City	0.23		0.21		0.21		.047	
**Female, >60 years**									
	Accra	0.28		0.21		0.09		.57	
	Bandung	0.57		0.54		0.16		.17	
	Ho Chi Minh City	0.00		0.00		N/A		N/A	
**Male, 12-17 years**									
	Accra	0.10		0.10		0.29		.09	
	Bandung	0.49		0.38		0.18		.11	
	Ho Chi Minh City	0.32		0.16		0.01		.96	
**Male, 18-24 years**									
	Accra	0.18		0.16		0.33		.01	
	Bandung	0.67		0.60		0.22		.01	
	Ho Chi Minh City	0.22		0.22		0.23		.05	
**Male, 25-59 years**									
	Accra	0.54		0.52		0.61		<.001	
	Bandung	0.67		0.67		0.70		<.001	
	Ho Chi Minh City	0.49		0.52		0.17		.10	
**Male, >60 years**									
	Accra	0.47		0.39		0.30		.01	
	Bandung	0.69		0.61		0.18		.06	
	Ho Chi Minh City	0.27		0.12		0.18		.57	

^a^N/A: not applicable.

**Figure 4:**
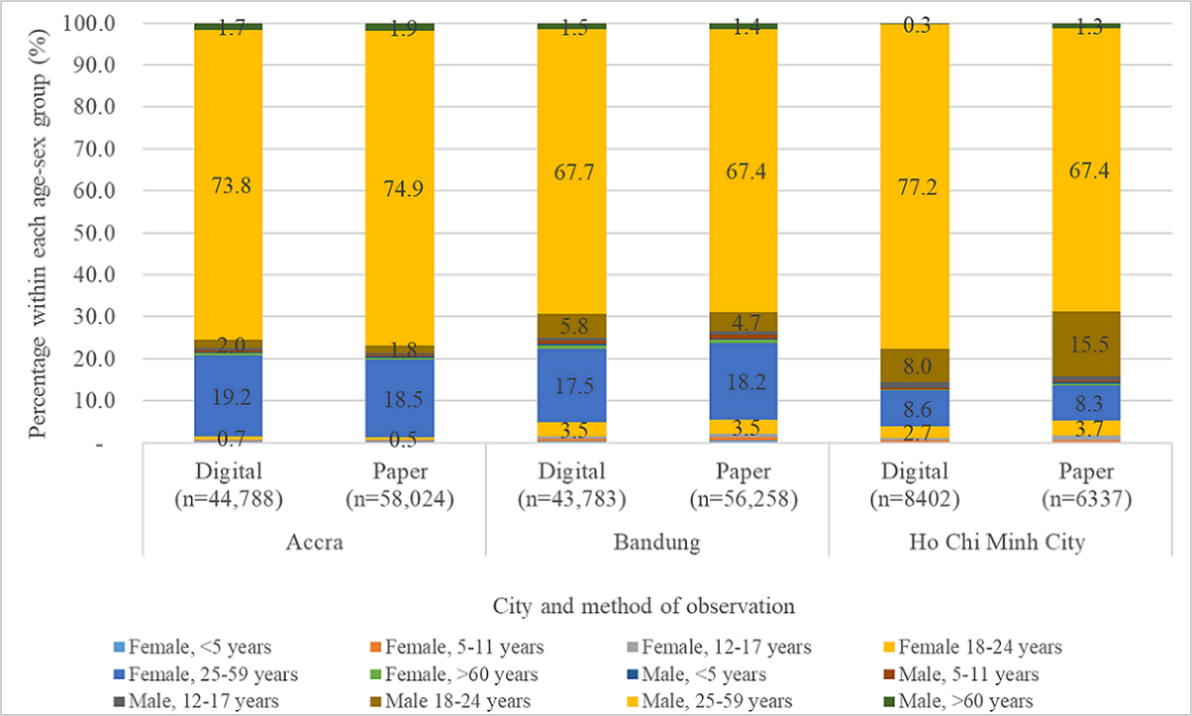
Four-wheeler occupants observed by age-sex group.

Among occupants of 4-wheeled vehicles, between two-thirds to three-fourths were adult males aged between 25 and 59 years (Figure 4). Generally, age-sex groups with low representation in the datasets, especially children, had lower reliability between digital and paper data collection methods, as did more narrowly defined age groups. For example, the proportion of 4-wheeler occupants that were females aged between 25 and 59 years was approximately 19% in Accra, 18% in Bandung, and 8% to 9% in HCMC; in those same cities, females aged between 12 and 17 years comprised 1% or less of 4-wheeler occupants, across digital and paper data collection methods (Figure 4). The correlation for seat belt use among females aged between 25 and 59 years was 0.73 in Accra, 0.58 in Bandung, and 0.04 in HCMC; the correlation for females aged between 12 and 17 years was 0.16 in Bandung, and was negative in Accra and HCMC.

**Figure 5:**
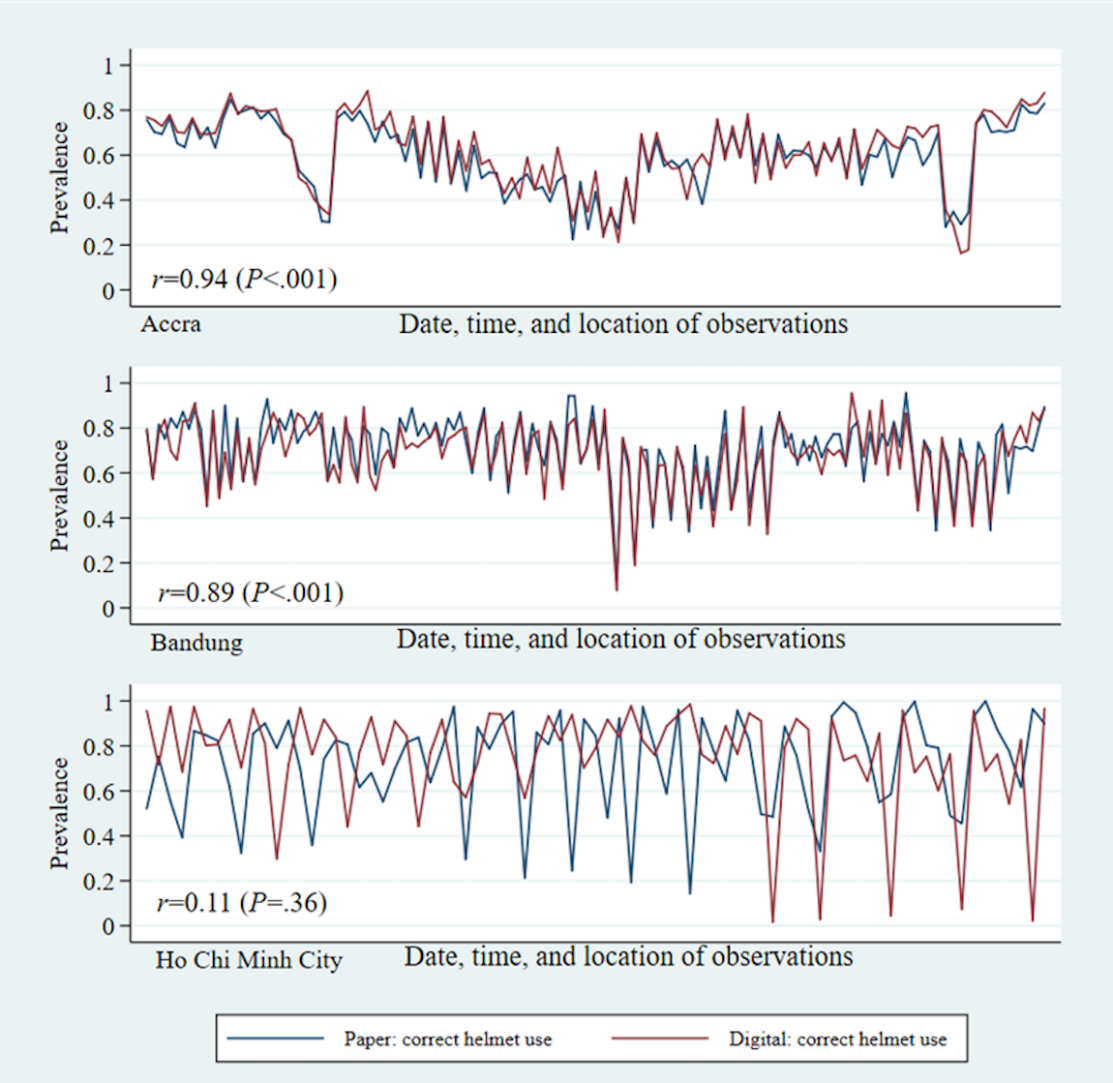
Prevalence of correct helmet use: reliability between digital and paper observations.

**Figure 6:**
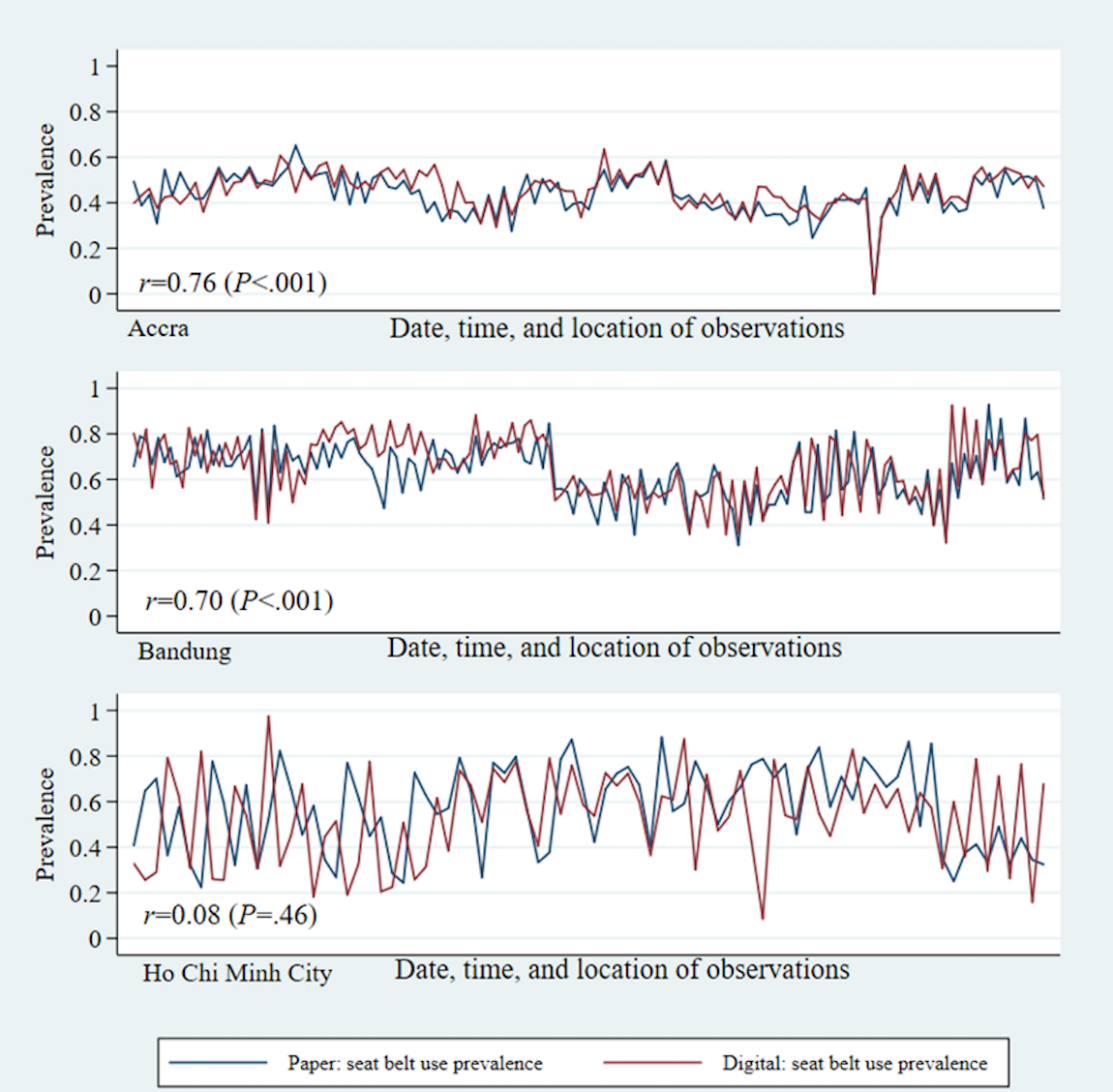
Prevalence of seat belt use: reliability between digital and paper observations.

**Figure 7:**
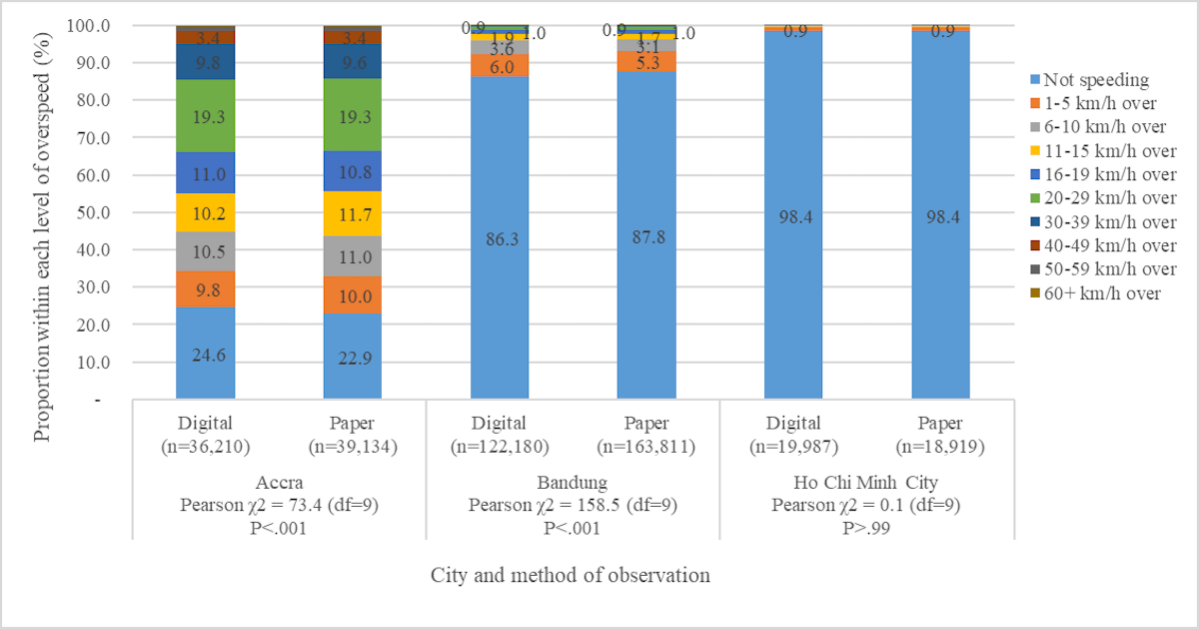
Prevalence of speeding by level of overspeeding and city: overall proportions and chi-square test of independence.

Pooling across all observation sessions of helmets and seat belts that could be matched between the 2 modalities, the digital and paper data collection methods, resulted in very similar risk factor prevalence, within only a few percentage points of each other except that of HCMC (Figures 5 and 6). Speeding observations also demonstrated overall consistency between the 2 formats, with HCMC demonstrating very high correlation between digital and paper data collection (Figure 7). In Bandung and Accra, the Pearson chi-square tests of independence showed statistically significant difference in the proportion of vehicles in different categories of overspeeding, although the actual percentages fall between 1 to 2 points from each other. Across cities, the prevalence of helmet use, seat belt use, and speeding and category of overspeeding was largely similar between digital and paper data collection methods (Table 4).

## Discussion

### Principal Findings

To our knowledge, this is the first study to evaluate the productivity and reliability of digital versus paper data collection in a roadside environment. This study also provides an illustration of different contexts and results from Accra, HCMC, and Bandung, using a standard methodology in all the sites. Each city had different number of observation sessions per risk factor, varying between 84 and 159 sessions depending on the risk factor and method. Considering only those digital and paper observation sessions matched by date, time, and location, the mean volumes of observations were statistically significantly higher for paper-based data than digitally collected data for all 3 risk factors in Accra and Bandung, and for helmet use in HCMC. However, larger sample size in paper data collection was not associated with meaningfully higher level of precision in forming prevalence estimates and same level of precision was achieved with relatively smaller sample size in DDC.

The differences in the number of observations per 90-min session between paper and digital methods varied by city, indicating that familiarity and dexterity varied by context. The data collection team in Accra showed the highest correlation and productivity across all risk factors. Bandung demonstrated high productivity with both data collection methods, with good reliability. In HCMC, the productivity of the DDC teams exceeded that of the paper data collection teams during the seat belt and speeding risk factor observational studies; however, the results showed moderate to low reliability in the prevalence of risk factors between the 2 methods. On inquiry, the reason behind this finding was the inadequate number of field staff, which led to the deviation from standard protocol. Although the protocol called for an observation team comprising separate observer and recorder, in HCMC the same person was observing and recording information. This resulted in overall low volumes and possibly negatively affected reliability.

There may be several reasons why the seat belt risk factor assessment showed lower reliability across the 3 cities, as compared with other risk factors. Although the reliability of correct helmet use was high in both Accra and Bandung, moderate reliability of seat belt observations in those cities might be offset by inability to capture accurate data on all the vehicle occupants. The seat belt use observations (in contrast to speeding observations) require the data collector to peer inside each vehicle; multiple research teams have reported difficulties with visibility into vehicles, particularly those with tinted windows [28]. In the helmet use observations, although the occupants of the motorcycle are visible, the data collector has to interpret whether a helmet is being worn correctly or not and may not have sufficient time to correctly assess all occupants. The helmet and seat belt use observations were more reliable for drivers than for passengers in both Accra and Bandung, and the reliability among the visible motorcycle passengers was higher than the less visible 4-wheeler passengers.

Similarly, in a moving vehicle it is sometimes difficult to document the age and sex of the occupants with certainty. This problem magnifies when occupants are young children or are wearing helmets, which could negatively impact the reliability of the observation. Furthermore, the seat belt observations required finer estimates of age group, which can lead to more chances for misclassification. However, because of the design of the observational study, the issue of misclassification by age or sex is minimized by the random assignments of the data collectors by date, time, location, team pairs, and digital versus paper data collection. These misclassifications are therefore randomly distributed across paper and digital format; however, despite random distribution, this likely also lowered the reliability when making comparisons by sex and age group. Misclassification of sex and age grouping is a general limitation of all studies based on roadside observations [28].

Another area where different observations were noted was the level of agreement on presence or absence of law enforcement. The presence and nature of police and camera enforcement, as well as environmental speed deterrents, must be same regardless of the method of data collection. The differences between observations might be simply a matter of timing (eg, if traffic police were at the location during only part of the session) or understanding of the environment (speed cameras vs closed circuit cameras; functional vs inactive speed cameras). These differences could be avoided by better training of the research assistants.

Speeding prevalences in paper and digital methods were found to be similar in HCMC and within 2 percentage points in Accra and Bandung, reflecting overall estimates as well as individual category of overspeeding. These small observed differences, though not meaningful, were in most instances statistically significant likely because of our very large sample sizes of vehicles and vehicle occupants. For instance, the prevalence of correct helmet use in Bandung was 0.68 according to the digitally collected dataset and 0.70 in the data collected through paper format. Although we do not judge 68% to be meaningfully different from 70%, with 173,043 observations on motorcycle occupants, this difference is statistically significant at the <.001 level of significance. The most important finding of this study was that despite the differences between digital and paper data collection formats in the volume of observations and variations in reliability, the overall prevalence of each risk factor was comparable. This finding is important for 2 reasons. First, switching from paper to DDC may reduce the mean number of observations per session, but it does not translate into a different prevalence of risk factors. DDC provided the advantage of reducing turnaround time, by eliminating the need of double data entry and cleaning required in paper format, which often delayed data analysis and dissemination of results. Second, the reliability of prevalence estimates for each risk factor obtained through digital method would allow to switch to DDC for future rounds of data collection in suitable environments, without impeding or distorting prior analysis of time trends for each road safety risk factor.

### Challenges

DDC was not completely error-free but was found to minimize data entry errors resulting from an extra data entry step [29]. Although automated skip patterns, mandatory fields, and logic checks support data completeness and accuracy, there were instances where these led to slow recording or incomplete information. For example, if research assistant initially recorded 4 car occupants but could only observe 3 as the vehicle moved on, the digital application was programmed to not allow the form to be uploaded without completing required information on all occupants. This issue was fixed by changing the required fields and making the form more flexible by adding a *nonobservable* option, in consideration of these extremely dynamic roadside environments. Misclassification error between genders and among age categories results in lower reliability between digital and paper methods, but as this error was random, it did not affect the risk factor prevalence in the whole samples.

Generally, DDC was well received in all 3 cities, but some challenges were identified by the DDC teams. First, the research assistants in Bandung found DDC to be tiring, especially in the upper back and neck areas because of prolonged rigid upper body position during information recording. This has been previously reported in other studies and this issue was resolved by limiting the number of sessions to 2 to 3 per day for each data collector [30]. Second, unstable network connections made it difficult for local teams to upload data, particularly in suburban road networks. Without being able to upload data and clear the tablet memory, the tablets slowed down, especially when research assistants had to conduct multiple back-to-back sessions. Third, the battery ran out quickly when the mobile network was used to upload data to the server; to tackle this issue, the data collectors were provided with backup batteries and power banks. There were at least 2 occasions where digital data were lost for the entire session; in one instance, the tablet malfunctioned and in the other, a research assistant ignored the prompt to save the completed forms after finishing the session. Fourth, research assistants reported that sometimes they recalled an error, such as misspecifying their location, only after uploading the data to the server; this recall error was handled by the data managers who corrected the error on the server. Fifth, although the data collection tool was uploaded bilingually in Bandung and HCMC, some research assistants recommended the use of visuals and photos for data entry as opposed to text-based drop-down menus. It was also recommended to have screens that could be scrolled down than swiped to improve the efficiency; to date, this function was not available in KoBoCollect app. It is important to note that most of the observed challenges could be addressed by training of field personnel, investing in good quality tablets and power backup, and further development of a user-friendly tablet interface.

### Implications for the Choice of Data Collection Method

Overall, paper-based data collection was found to be more productive method for observational studies in roadside environment. A possible explanation for this finding is that writing on paper is easier or at least more familiar initially; typing using an onscreen keyboard might be slower in some circumstances, owing to the requirement of entering information on each individual vehicle or vehicle occupant separately. This could be initially challenging for the average data collector, particularly if they were not familiar with Android technology or had not used a mobile phone or tablet on a regular basis. There appeared to be 2 learning curves for data collectors when moving to an electronic format; not only must they develop familiarity with the data entry system, but also with the content of the survey form as displayed in an electronic format [29]. The learning curve to use an electronic data entry system is usually proportionate to the degree of complexity in the electronic format and length of time spent developing experience. Considering our observational forms were much shorter and less complex (relative to a typical household survey), the time advantages of DDC might be less pronounced using an initially unfamiliar technology.

In those circumstances where sheer productivity is not the central focus and precision of estimates could be maintained by comparatively smaller sample sizes, DDC may be preferred, as shown in this study. DDC provides the advantages of standardization; logic checks; immediate updating of questionnaire version without wasting previously printed material; automatic synchronization of metadata, pictures, and GPS coordinates with the correct survey; and both remote and real-time monitoring of data quality, as documented by other researchers as well [14,15]. DDC requires a one-time cost of mobile or tablet devices as well as the cost of setting up a server and designing the digital module. Other longer-term costs include maintenance of devices, data plans, or internet service. Therefore, in some environments, paper-based data collection might be more feasible. However, in settings and circumstances where these conditions could be met, DDC could cut the time to aggregate large datasets, reduce the cost related with printing, transporting, and storing paper questionnaires, double data entry, reconciliation through hard copy checks, and associated human resources. The relative cost and availability of human and material resource could also impact the choice of data collection method.

### Limitations

This paper does not directly address the efficiency of digital versus paper data collection. As mentioned earlier, efficiency of a data collection system is contextually determined and may imply time and cost efficiency or could be tied to logistical feasibility in a given environment. This study did not collect information on differential cost of supplies, equipment, training, human resource, data collection, and management.

### Conclusions

DDC provides a reliable and convenient means for conducting large volume roadside observational studies of behavioral risk factors and reducing the turnaround time from data collection to policy decisions. There is some site-related variability in implementing DDC, but the big-picture results are comparable with the paper-based approach. There are upfront costs associated with resources to program the digital applications and acquire the necessary equipment for digital data collection, but the benefits of automating future rounds of data collection with quality data may help in reducing turnaround time and thus prove beneficial in the long run.

## Abbreviations

BIGRS: Bloomberg Initiative for Global Road Safety
DDC: digital data collection
GPS: Global Positioning System
HCMC: Ho Chi Minh City
LMIC: low- and middle-income country
mHealth: mobile health
RTI: road traffic injury
